# Transcutaneous electrical acupoint stimulation for stage 1 hypertension: protocol for a randomized controlled pilot trial

**DOI:** 10.1186/s13063-020-04493-x

**Published:** 2020-06-22

**Authors:** Zhong-Xue Tian, Cun-Zhi Liu, You-Sheng Qi, Jian-Feng Tu, Ying Lin, Yu Wang, Jing-Wen Yang, Guang-Xia Shi, Jun-Hong Liu, Li-Qiong Wang

**Affiliations:** 1 Acupuncture Research Center, School of Acupuncture-Moxibustion and Tuina, Beijing University of Chinese Medicine, No. 11, Bei San Huan Dong Lu, Chaoyang District, Beijing, 100029 China; 2 Nanyuan Community Health Service Center, Fengtai District, Beijing, China; 3 Department of Acupuncture and Moxibustion, Beijing Hospital of Traditional Chinese Medicine Affiliated to Capital Medical University, Dongcheng District, Beijing, China

**Keywords:** Transcutaneous electrical acupoint stimulation (TEAS), Lifestyle education, Stage 1 hypertension, Randomized controlled trial

## Abstract

**Background:**

Hypertension is a major pathogenic factor of cardiovascular diseases. Insufficient blood pressure control rate and sub-optimal medication adherence remain challenges for effective management of hypertension. Transcutaneous electrical acupoint stimulation (TEAS) has been used to treat various diseases, including hypertension, but the scientific evidence for its benefit remains insufficient. Therefore, we will perform a randomized, controlled clinical trial in patients with stage 1 hypertension to evaluate the effect of TEAS.

**Methods/design:**

The study will be a two-arm parallel, randomized controlled trial. Sixty patients with stage 1 hypertension will be randomly assigned to the TEAS group and the control group in a 1:1 ratio. The participants in the TEAS group will receive non-invasive acupoint electrical stimulation for 30 min at four acupoints in the upper and lower extremities at home, 4 times weekly for 12 weeks for a total of 48 sessions. Participants in the control group will not receive any form of acupoint stimulation. All participants in both groups will receive lifestyle education on how to control high blood pressure, including diet, weight control, and exercise. The primary outcome measure will be the change of the mean systolic blood pressure from baseline to 12 weeks. Secondary outcomes include the change of mean diastolic blood pressure, quality of life, body mass index, and physical activity level.

**Discussion:**

This pilot, randomized, controlled trial will explore the feasibility of TEAS. It will also provide potential clinical evidence for the efficacy and safety of TEAS in the treatment of patients with stage 1 hypertension. The results of this study will be published in peer-reviewed journals. Furthermore, this pilot trial as the precursor of a large scale randomized controlled trial will inform the sample size of the subsequent trial.

**Trial registration:**

Chinese clinical trial registry, ChiCTR1900025042, Registered on 8 August 2019 (http://www.chictr.org.cn/showproj.aspx?proj=41496).

## Background

Hypertension is a major risk factor for cardiovascular disease death and its global burden is increasing [1]. Stage 1 hypertension is a systolic blood pressure (SBP) of 140–159 mmHg or diastolic blood pressure (DBP) of 90–99 mmHg and is one of the common subtypes of hypertension in China [2]. Previous studies have shown that a 10-mmHg reduction in SBP or a 5-mmHg reduction in DBP significantly reduces the relative risk of coronary heart disease [3], and blood pressure (BP) control in patients with stage 1 hypertension below 140/90 mmHg also significantly reduced the risk of stroke or death [4]. Therefore, it is necessary to the adequately control BP in patients with stage 1 hypertension.

At present, the treatment of hypertension includes the use of antihypertensive drugs and lifestyle interventions [5]. A systematic review showed that drug treatment is ineffective for many patients with stage 1 hypertension [6]. Therefore, widely used lifestyle changes, including weight loss, reduced sodium intake, regular physical exercise, smoking cessation, and moderate alcohol consumption, are also important [7, 8]. However, the benefits of lifestyle interventions on blood pressure will decrease over time [9, 10]. Therefore, a sustainable and effective long-term non-drug treatment is needed [11].

Acupuncture is an important part of traditional Chinese medicine therapy and has proven antihypertensive effect in hypertensive patients [12–15]. However, some patients worry about the economic cost of acupuncture or the time it takes to get to the hospital to have it [16, 17]. Transcutaneous electrical acupoint stimulation (TEAS) is a new non-invasive acupuncture treatment, which combines the effects of transcutaneous electrical nerve stimulation (TENS) with acupoint stimulation [18]. It stimulates the afferent nerves with low-voltage pulses transmitted through the skin at an acupoint, and TEAS may have a better antihypertensive effect than traditional acupuncture. In a study, Jacobsson et al. found that TEAS may have additional antihypertensive effect on 24-h ambulatory BP in those patients who did not respond to pharmacological treatment [19]. But it had some limitations, including the low quality of the study design and inadequate control groups. There are no randomized controlled trials to evaluate the efficacy of TEAS in the treatment of stage 1 hypertension at the present time.

In this study, we hypothesize that TEAS has an additional antihypertensive effect on stage 1 hypertension patients after lifestyle interventions. The results derived from this study will be used to explore the efficacy and safety of TEAS in lowering BP and calculated the appropriate sample size for a future large clinical trial.

## Methods/design

### Study design

This open-label, two-armed, randomized, controlled pilot trial will be conducted at Nanyuan Community Health Service Center which is one of chronic disease management bases in Beijing, China. The protocol has been registered on Chinese clinical trial registry (No ChiCTR1900025042) and approved by the ethics committee of Beijing University of Chinese Medicine (No 2019BZHYLL0208). The protocol follows the Declaration of Helsinki and will be reported in accordance with the Standard Protocol Items (SPIRIT) (Additional file 1).

### Study setting, recruitment

**Fig. 1:**
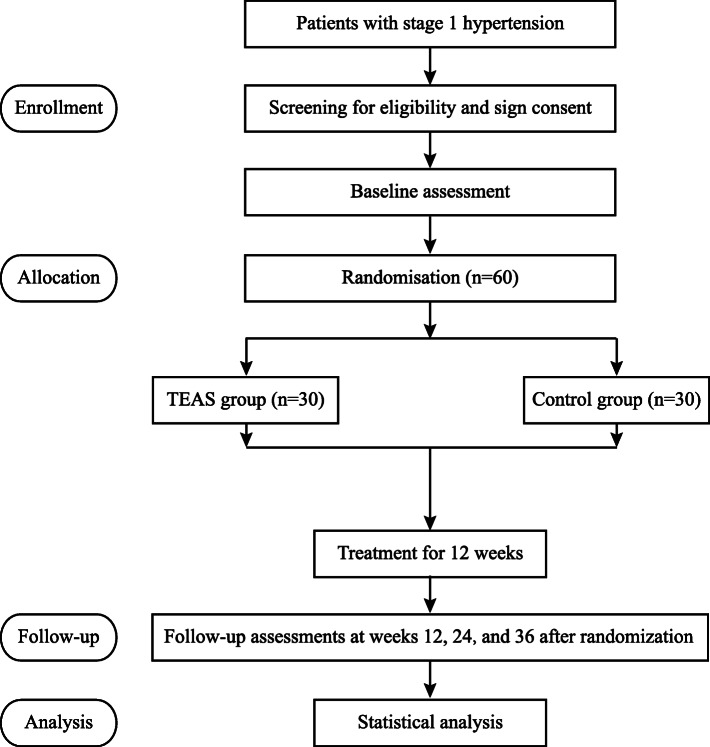
Flow diagram

The clinical research coordinator will recruit 60 patients with stage 1 hypertension at Nanyuan Community Health Service Center in Beijing. The main recruitment strategies include the community’s chronic disease management software, WeChat advertisements, and on-site flyers at the outpatient department. The clinical research coordinator will conduct preliminary information screening by telephone or face to face. Then, patients who pass the preliminary screening will undergo a blood pressure test to determine whether they will be finally included. Written informed consent will be obtained by the clinical research coordinator at the outpatient department before randomization. Figure 1 shows the flow diagram of the trial.

### Participants

#### Inclusion criteria

1. Aged between 35 and 65 years (male or female)
2. Fulfilling the diagnostic criteria for stage 1 hypertension (SBP of 140–159 mmHg and/or DBP of 90–99 mmHg) [20]
3. Capacity to consent to taking part in the study and complete the questionnaires.
4. Voluntary signing of informed consent

#### Exclusion criteria

1. Contraindications for the use of acupoint stimulators: wearing a pacemaker and other implanted medical devices; suffering from acute diseases, infectious diseases, malignant tumors; cardiovascular disease, cerebrovascular disease, liver and kidney dysfunction, or other malignant diseases; scars, bruises, scratches, or inflammation on the skin of the acupuncture points.
2. Secondary hypertension caused by other vascular diseases, primary aldosteronism, other endocrine hypertension, etc.
3. Receiving drugs that affect blood pressure except antihypertensive drugs in previous 2 months such as glucocorticoids, central nervous system inhibitors.
4. Uncontrolled diabetes.
5. Drug or alcohol abuse.
6. Pregnant women or women in lactation period.
7. Acupuncture treatment within the past month.
8. Participation in another clinical study in the past month.

### Randomization and masking

Eligible patients will be randomly assigned to the TEAS group or control group in a ratio of 1:1. The randomization sequence will be generated with the software SAS 9.3 by an independent statistician (LQ. Wang, Beijing University of Chinese Medicine, China) using block randomization. Randomization sequence will be stored by a non-involved investigator. When an eligible patient needs to be randomized, the clinical research coordinator will contact the non-involved investigator by phone to obtain random number and assign him/her to one group. The design is open label, but the outcome assessors and statisticians who are blinded to group allocation will be responsible for collecting and analyzing the data, respectively.

### Interventions

The physician will provide lifestyle education to all patients for 12 weeks, which mainly includes sending relevant antihypertensive information to patients every week and holding health lectures every month. The content of the information includes influence of lifestyle on hypertension control, especially the strength of dietary health, and the importance of moderate physical exercise, maintaining normal weight and quitting smoking. In addition, we will establish a communication group to answer questions raised by patients during the intervention. If patients were on antihypertensive medication, they were instructed to continue this medication but to abstain from changing the type or dosage of drugs.

#### Control group

Subjects randomized to the control group will not be given TEAS treatment except the lifestyle education.

#### Transcutaneous electrical acupoint stimulation group

**Fig. 2:**
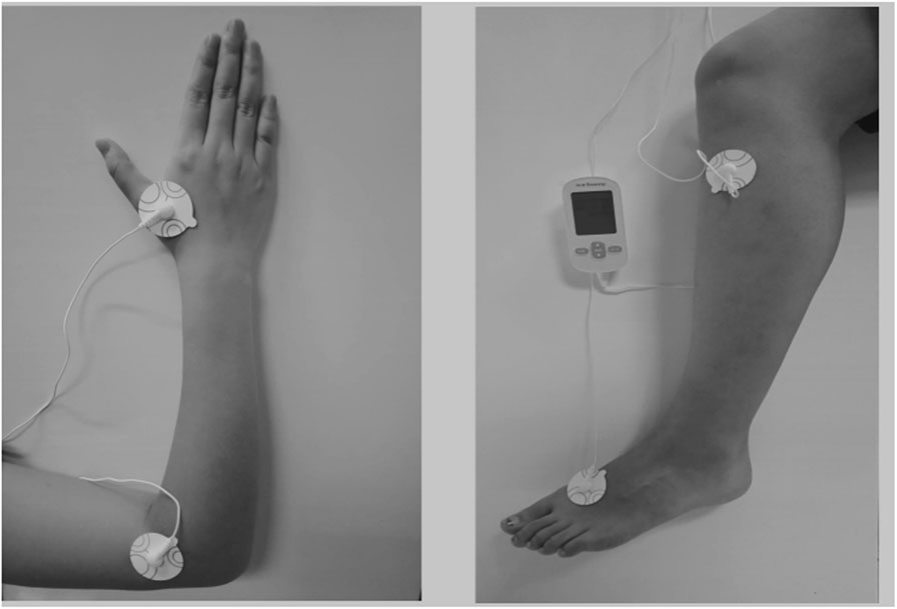
Locations of acupoints

In addition to the lifestyle education, the participants in this group will receive TEAS 48 sessions over a period of 12 weeks (4 sessions/week) at home. TEAS will be performed using a portable instrument for low-frequency electrotherapy (SDP-330, Yuwell, Suzhou Medical Appliances Co, Ltd., Suzhou, China). The device is a so-called dual channel stimulator with two pairs of rubber electrodes (30 mm × 30 mm) applied to the skin as illustrated in Fig. 2. The instrument has 8 different stimulus modes; patients will be asked to select a fixed mode (“press” or “knock” or “knead” mode) throughout the trial. It has ten different stimulus intensities, and patients could choose based on their tolerance.

According to the previous studies [21, 22], bilateral Hegu (LI4), Quchi (LI11), Zusanli (ST36), and Taichong (LR3) will be selected in our trial. At the first session, the electrodes will be placed at the ipsilateral LI4 and LI11, and then the appropriate stimulus intensity will be selected according to tolerance of each individual for 15 min after connecting the instrument, then the opposite limb will be treated in the same way for 15 min. In the second session, the ST36 and LR3 acupoints of the leg will be treated for 15 min in the same way. Two pairs of acupoints will be stimulated alternatively every other day. Locations of acupoints are shown in Table 1.

**Table 1:** Locations of acupoints in the TEAS group

Acupoints	Locations^a^
Hegu (LI4)	On the dorsum of the hand, radial to the midpoint of the second metacarpal bone.
Quchi (LI11)	On the lateral aspect of the elbow, at the midpoint of the line connecting LU5 with the lateral epicondyle of the humerus.
Zusanli (ST36)	On the anterior aspect of the leg, on the line connecting ST35 with ST41, 3 B-cun inferior to ST35. ST36 is located on the tibialis anterior muscle.
Taichong (LR3)	In the depression anterior to the junction of first and second metatarsal bones.

^a^1 cun (≈ 20 mm) is defined as the width of the interphalangeal joint of patient’s thumb

The acupuncturist will train the participants on how to locate the acupoints and use the instrument face-to-face. They will also send paper instructions and acupoint maps to help patients with TEAS treatment at home. The patients will be required to record the date, the time, and the specific acupoints when using the instrument. The instrument will be given to the patients when they have completed the prescribed treatment times and visits.

### Outcomes

#### Primary outcome

The primary outcome of the study is the change in mean SBP between baseline and 12 weeks after treatment. BP will be measured and recorded by using an electronic sphygmomanometer (HEM-7136, Omron Corporation, Kyoto, Japan) in both groups. Patients were asked to rest quietly for at least 5 min before taking blood pressure measurements of the upper arm in the sitting position, with the upper arm at heart level [23]. Blood pressure in both upper arms should be measured at the first test; the side with the higher blood pressure reading will be selected. At subsequent detection time points, the measurement will be repeated 3 times every 5 min; the average value of the last 2 readings will be taken as the mean BP [24].

#### Secondary outcomes

The mean SBP will be evaluated at weeks 24 and 36 after randomization. The mean DBP, body mass index (BMI), International Sports Activity Questionnaire (IPAQ), and Short Form (SF-12) will be evaluated at baseline, 12 weeks, 24 weeks, and 36 weeks. The DBP measurement method is consistent with SBP. BMI is weight (kg) divided by the square of height [25, 26]. The IPAQ questionnaire includes the frequency, intensity, timing, and type of exercise [27], which will be divided into three levels of physical activity levels by metabolic equivalent (MET). The SF-12 is a questionnaire to evaluate general health outcomes including psychological and physical domains [28, 29].

**Fig. 3:**
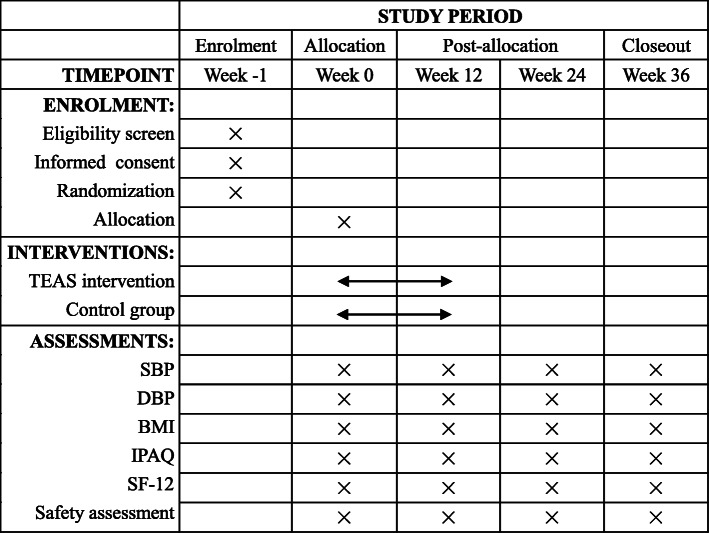
Schedule of enrolment, intervention, and assessments (SPIRIT) figure

The schedule of enrolment, intervention, and assessments is shown in Fig. 3.

### Adverse events

Any adverse events will be reported by the patients and outcome assessors, including severe pain, local infection, or unbearable tingling during treatment, and will be recorded during the trial. If any serious adverse events or uncontrolled hypertension occur, they will be immediately reported to the primary investigator and the patient will be withdrawn from the trial. There is no anticipated harm and compensation for trial participation.

### Quality control

The program has been reviewed and revised by clinical acupuncturists and statisticians. Prescribed standard operating procedures are screening of patients, blood pressure monitoring, therapeutic criteria, random methods, and acupoint stimulation, and the case report form (CRF) will be filled in. The CRF will be completed on paper copies and then entered into the Excel spreadsheet to ensure the accuracy of the data. All modifications of the data can be traced through the CRF. The ethics committee of Beijing University of Chinese medicine will audit trial conduct per 12 months.

### Sample size

This is a pilot trial, and the sample size is based on the minimum sample size of clinical trials. Each group will include 30 participants, a total of 60 participants. This is the minimum number of participants used for comparative analysis to determine the initial data of main outcome measurements and explore for future large-scale randomized controlled trial [30].

### Statistical analysis

Statistical analysis will be conducted by SPSS version 23.0 (IBM SPSS Statistics, NY, USA). Intention-to-treat analysis (ITT) will be applied to all cases randomly assigned to each group [31]. We will try our best to collect the data for the patients who quit or lost the visit in this trial. The last observation carried forward (LOCF) will be used for missing data. There is no interim analysis or additional analysis in this trial. The level of significance will be established at *α* < 0.05 with a two-sided test. Patients’ baseline characteristics will be described for the two groups. Continuously distributed variables will be described using mean ± standard deviation (M ± SD) or median and quartile intervals. Discrete variables will be described by frequencies and percentages. For the primary outcome, the change in SBP between baseline and 12 weeks after treatment will be calculated and the difference of the change in SBP between the two groups will be tested using Student’s *t* test or the Wilcoxon rank sum test. For secondary outcomes, Student’s *t* test, chi-squared test, Fisher’s exact test, or the Wilcoxon rank sum test will be used to test the difference of the outcomes including the DBP, BMI, IPAQ, and SF-12, between groups according to the distribution of variables.

## Discussion

The awareness, treatment, and control of hypertension are insufficient in China [32]. For early hypertension, the symptoms are inconspicuous so that most patients pay little attention to the condition and may ultimately develop severe hypertension, thus increasing the risk of cardiovascular diseases [33]. In addition to the financial burden, drug treatment has potential side effects [34]. For patients with high blood pressure who refuse to take medicine or have low drug compliance, other effective methods of lowering blood pressure need to be found.

It has been shown that acupuncture is an effective way to lower blood pressure [35, 36]. As a non-invasive treatment, TEAS is similar to acupuncture in stimulating acupoints, and many clinical studies have shown the feasibility and safety of TEAS [37–39]. This approach to intervention has several advantages. First, the self-adjusting mode of stimulation parameters allows patients to adjust the frequency and intensity of treatment according to their own endurance capacity to prevent skin damage or other adverse reactions. Second, patients can use it at home at any time, which greatly saves time and economic cost. The study will be conducted within 12 weeks to observe the long-term effects of TEAS on patients with stage 1 hypertension.

In addition to feasible interventions, the use of an appropriate control group is a key issue in designing clinical trials. In mild hypertension, lifestyle change is a necessary step to lower blood pressure [40]. We will test whether TEAS combined with lifestyle education has a greater effect on lowering blood pressure and controlling conditions in patients with stage 1 hypertension. To minimize the confounding effects, the two groups will receive scientific information about hypertension lifestyle interventions at the same time. During the trial, we will call or send messages to encourage patients in the TEAS group to adhere to the instrument treatment to improve compliance.

The limitation of our trial is that the patients are not blinded. Based on the nature of chronic disease and barrier of time necessary for frequent transportation to hospital, the majority of treatment will be operated by patients themselves at home. And patients can distinguish whether the instrument is on or not. It is difficult to blind patients. Moreover, this pilot trial is the precursor to a large scale randomized controlled trial in which the aim is to evaluate the effectiveness not the efficacy of transcutaneous electrical acupoint stimulation as adjunctive therapy for stage 1 hypertension in the real world. Hence, we do not set sham therapy as the control group.

In conclusion, we hypothesize that the systolic blood pressure in the TEAS group will be lower than that in the control group after 12 weeks of treatment, and the results will allow patients to delay the use of hypertensive drugs, prevent patients from becoming seriously hypertensive hypertension, and provide evidence for future large clinical trials. Our results will be published in peer review journals in the form of articles.

### Trial status

Protocol: version 2.0, 10 June 2019.

The first patient was recruited on 4 September 2019. At the present time, a total of 30 patients had been randomized. The final date of follow-up is expected to be October 2020. This protocol was submitted prior to the recruitment of total 30 patients.

## Supplementary information

**Additional file 1.** Completed Standard Protocol Items: Recommendation for Interventional Trials (SPIRIT) 2013 Checklist: items addressed in this clinical trial protocol.

## Data Availability

All of the individual participant data collected during the trial after de-identification will be available for anyone who wishes to access the data immediately following publication. Any data required to support the protocol can be supplied on request.

## Abbreviations

TEAS: Transcutaneous electrical acupoint stimulation
TENS: Transcutaneous electrical nerve stimulation
BP: Blood pressure
BMI: Body mass index
SBP: Systolic blood pressure
DBP: Diastolic blood pressure
CRF: Case report form
SF-12: 12-Item Short Form Health Survey
ITT: Intention-to-treat analysis
LOCF: Last observation carried forward
